# Prevalence and risk factors for falls among older Chinese adults in the community: findings from the CLHLS study

**DOI:** 10.1590/1414-431X2024e13469

**Published:** 2024-05-17

**Authors:** Haodong Liang, Zijie Zhang, Haitian Lai, Tianzhao Tian

**Affiliations:** 1 The Affiliated TCM Hospital of Guangzhou Medical University, Guangzhou, China; 2 The Second Clinical Medical College of Guangzhou University of Chinese Medicine, Guangzhou, China; 3 The Eighth Clinical Medical College of Guangzhou University of Chinese Medicine, Guangzhou, China

**Keywords:** Falls, Prevalence, Risk factors, Older adults, China

## Abstract

Older adults have a high prevalence of falls due to a decline in physiological functions and various chronic diseases. This study aimed to investigate the prevalence of and risk factors for falls among older individuals in the Chinese Longitudinal Healthy Longevity Survey (CLHLS). We collected information from 9737 older individuals (average age=84.26 years) from the CLHLS and used binary logistic regression analysis to explore the independent risk factors and protective factors for falls. The logistic regression analysis results are reported as adjusted odds ratios (aORs) and 95% confidence intervals (95%CIs). The prevalence of falls among older adults in China was 21.6%, with women (24.6%) having a higher prevalence than men (18.1%). Logistic regression analysis revealed that never (or rarely) eating fresh fruit, difficulty with hearing, cataracts, and arthritis were the common independent risk factors for falls in older Chinese men and women. Among men, age ≥80 years (aOR=1.86), never doing housework (aOR=1.36), and dyslipidemia (aOR=1.47) were risk factors, while eating milk products once a week was a protective factor. Alcohol consumption (aOR=1.40), physical labor (aOR=1.28), and heart disease (aOR=1.21) were risk factors for falls in women, while a daily sleep duration of 6-12 h and garlic consumption once a week were protective factors. The prevalence of falls among older adults in China is 21.6% and is greater in women than in men. These risk and protective factors can be used to formulate reasonable recommendations for living habits, diet, and chronic disease control strategies.

## Introduction

Falls have been described as falling to the ground or a lower level outside the expected range, excluding falls caused by external force, loss of consciousness, epilepsy, etc. (1). There is a high prevalence of falls in older adults due to a decrease in various physiological functions and chronic diseases. Approximately 25.9% of older adults in the Lleida community of Spain fall at least once a year, and 70% of those adults fall repeatedly (2,3). Falls can cause many negative effects, including fractures, cerebral hemorrhage, decreased mobility, anxiety, depression, and fear, which not only affect the quality of life of older adults but also cause heavy mental and economic burdens for families and society (4,5). In the United States, falls have become the sixth leading cause of death for people older than 65 years, and medical expenses related to falls have exceeded 50 billion US dollars (6). In China, the direct medical cost associated with falls is approximately 5 billion RMB, while the socioeconomic cost is 60-80 billion RMB (7). According to data from China's seventh national population census in 2020, the proportion of people ≥65 years old was 13.50%, up from 8.87% in the sixth census in 2010 (8). With the acceleration of the aging process, the problem of falls among older adults will become more serious in the future.

Previous studies have shown that advanced age, heart disease, arthritis, and cognitive impairment are risk factors for falls (9), but recent research on the epidemiology of falls in China and the impact of diet and lifestyle habits on falls in older Chinese men and women is still lacking. To enable the community and medical systems to formulate targeted interventions to prevent falls among older adults, our research explored the prevalence and risk factors for falls among older Chinese men and women based on the Chinese Longitudinal Healthy Longevity Survey (CLHLS) (2018 wave) data released in 2018. We hope that this study can provide a decision-making basis for reducing the risk of falls in older adults and the economic burden of disease caused by falls.

## Material and Methods

### Study design and population

The data used in this study were obtained from the CLHLS (2018 wave), and the recruitment of patients occurred from 2017 to 2018 (https://doi.org/10.18170/DVN/WBO7LK). The CLHLS studied social, behavioral, environmental, biological, and other factors affecting health and longevity and their interaction mechanisms. The survey scope of the CLHLS covered 23 provinces, cities, or autonomous regions in China, and nearly 50% of the cities or counties in these provinces were randomly selected for the survey (10), which shows that the research data are representative. The design and sampling details of the CLHLS can be found in previously published literature (11). The survey content of the CLHLS included information on older adults' health self-assessment, eating habits, behavior and lifestyle, living environment, economic status, and other factors. The CLHLS data released in 2018 surveyed 15,874 people older than 65 years, all of whom were living in the community.

### Ethical approval

The design and implementation of the CLHLS were approved by Duke University (USA) and the Peking University Research Ethics Committee (China, IRB00001052-13074), and all participants or their agents provided written informed consent.

### Outcomes

In this study, falls were defined as sudden and unintentional body position changes occurring on the same plane or a plane lower than the starting position (12), and the outcome variable was whether the patient fell in the past year (a binary score of ‘yes' or ‘no').

### Potential risk factors

The potential risk factors addressed in this study included sociodemographic information, living habits, eating habits, and health status.

Sociodemographic data included age, sex, body mass index (BMI), residential area (city, town, or rural), coresidence (with household member, alone, or in an institution), public old-age insurance (no *vs* yes), waist circumference, and hip circumference. BMI was calculated as weight (kg)/height^2^ (m^2^). The individuals were divided according to their BMI into the following groups: obesity (≥28 kg/m^2^), overweight (24 to 27.99 kg/m^2^), normal (18.5 to 23.99 kg/m^2^), and underweight (<18.5 kg/m^2^) (13).

The participants' living habits included sleep duration (hours), smoking status (no *vs* yes), alcohol consumption status (no *vs* yes), and frequency of physical labor, housework, tai chi chuan, square dancing, and garden work (participants provided answers based on their own living habits).

Eating habits included the frequency of eating fresh fruit, vegetables, sugar, garlic, milk products, and nut products and the type of water consumed (boiled or unboiled water) (participants provided answers based on their own eating and drinking habits).

The health conditions included difficulty with hearing, hypertension, diabetes, heart disease, tuberculosis, cataracts, glaucoma, arthritis, dementia, dyslipidemia, chronic nephritis, and hepatitis. The diagnosis of the above chronic diseases was self-reported by the participants or their proxies.

### Data collection

**Figure 1:**
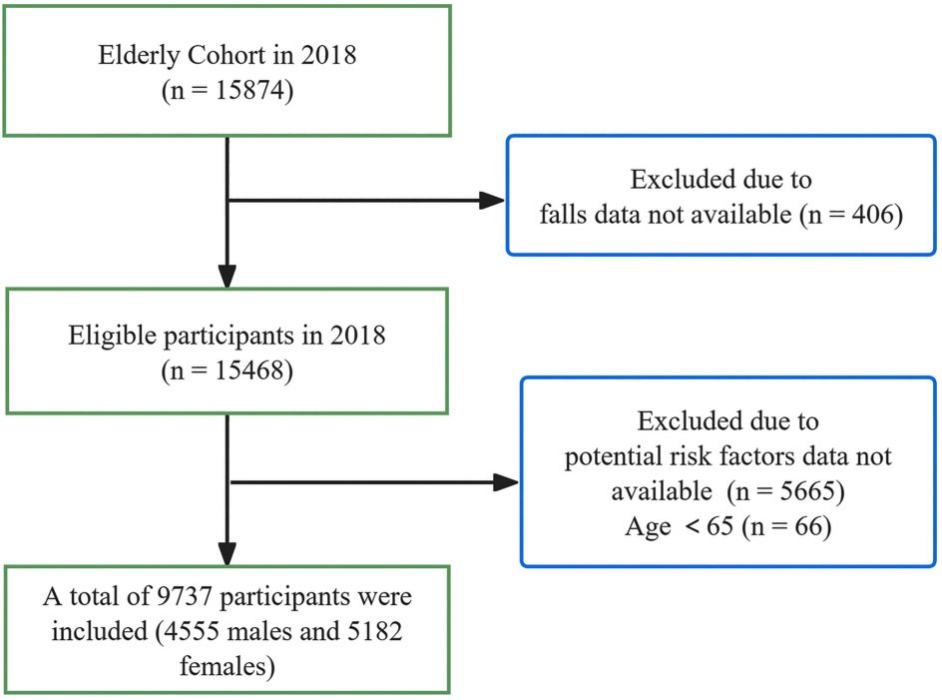
Flow chart of the study sample enrollment process (n=9737).

The data of this study was from CLHLS, which was issued by the Research Center for Healthy Aging and Development at Peking University in 2018. To ensure the accuracy of the research results, this study excluded participants who had not filled in the questions related to the dependent and independent variables. In addition, if the participants answered, “do not know”, “cannot answer”, or other vague answers to questions about the variables concerned in this study, then they were also treated as having missing data. The sample screening process used in this study is shown in Figure 1.

### Statistical analysis

We used SPSS 24.0 (IBM, USA) for the data analysis. Counting data are reported as the frequency and composition ratio and were analyzed by the chi-squared test (or Fisher's exact test). The measurement data are reported as means±SD and were compared between groups by independent-samples *t*-tests. The nonparametric Mann-Whitney U test was used for ranked data. We used a logistic regression method to conduct a multivariate analysis for all independent variables that were significantly different according to the univariate analysis. Multivariate logistic regression was used to calculate adjusted odds ratios (aORs) and 95% confidence intervals (CIs). P<0.05 was considered to indicate statistical significance.

## Results

### Prevalence of falls

A total of 9737 people ≥65 years old were included in this study (4555 males and 5182 females). The statistical results showed that the prevalence of falls among older adults in China was 21.6% (2101/9737). To further understand the prevalence of falls among older adults in terms of age, sex, BMI, type of residence, and the number of people living together, we calculated the prevalence of falls in different subgroups. The prevalence of falls among 65-69-, 70-79-, and ≥80-year-olds was 14.2, 17.3, and 24.8%, respectively, indicating that the older the individuals are, the greater the prevalence of falls. The prevalence of falls in men and women was 18.1 and 24.6%, respectively. The prevalence of falls in underweight older adults was the highest (25.6%), while the prevalence in overweight older adults was the lowest (19.0%). The prevalence of falls among older adults living in cities was 22.6%, while that among those living in rural areas was 21.2%. The prevalence of falls among older adults living with family members was 21.1%, which was significantly lower than the 26.2% reported in older care institutions. The statistical results for fall prevalence are shown in Table 1.

**Table 1:** Prevalence of falls in older adults from the Chinese Longitudinal Healthy Longevity Survey (CLHLS) (2018 wave) according to subgroups.

Variables	Total (n)	Number of Fallers	Prevalence (%)
	9737	2101	21.6
Age			
<70	1091	155	14.2
70-79	2590	447	17.3
≥80	6056	1499	24.8
Gender			
Male	4555	826	18.1
Female	5182	1275	24.6
BMI			
<18.5	1566	401	25.6
18.5-23.99	4998	1080	21.6
24-27.99	2347	446	19.0
≥28	826	174	21.1
Residential area			
City	2167	489	22.6
Town	3263	697	21.4
City and town	5430	1186	21.8
Rural	4307	915	21.2
Co-residence			
With household member(s)	7884	1662	21.1
Alone	1586	369	23.3
In an institution	267	70	26.2

### Baseline characteristics

A total of 9737 older people were included in this study, with an average age of 84.26±11.4 years. We have provided detailed statistical analysis of the sociodemographic information, living habits, eating habits, and health status of the older adults (Supplementary Table S1). To validate the conclusions of this study more accurately and practically, we analyzed the potential risk factors for falls in older adults stratified by sex (men and women). All statistically significant variables in the univariate analysis (Supplementary Table S1) were included in the multivariate logistic regression model for further analysis (Supplementary Table S2).

### Risk factors for falls in older men

**Figure 2:**
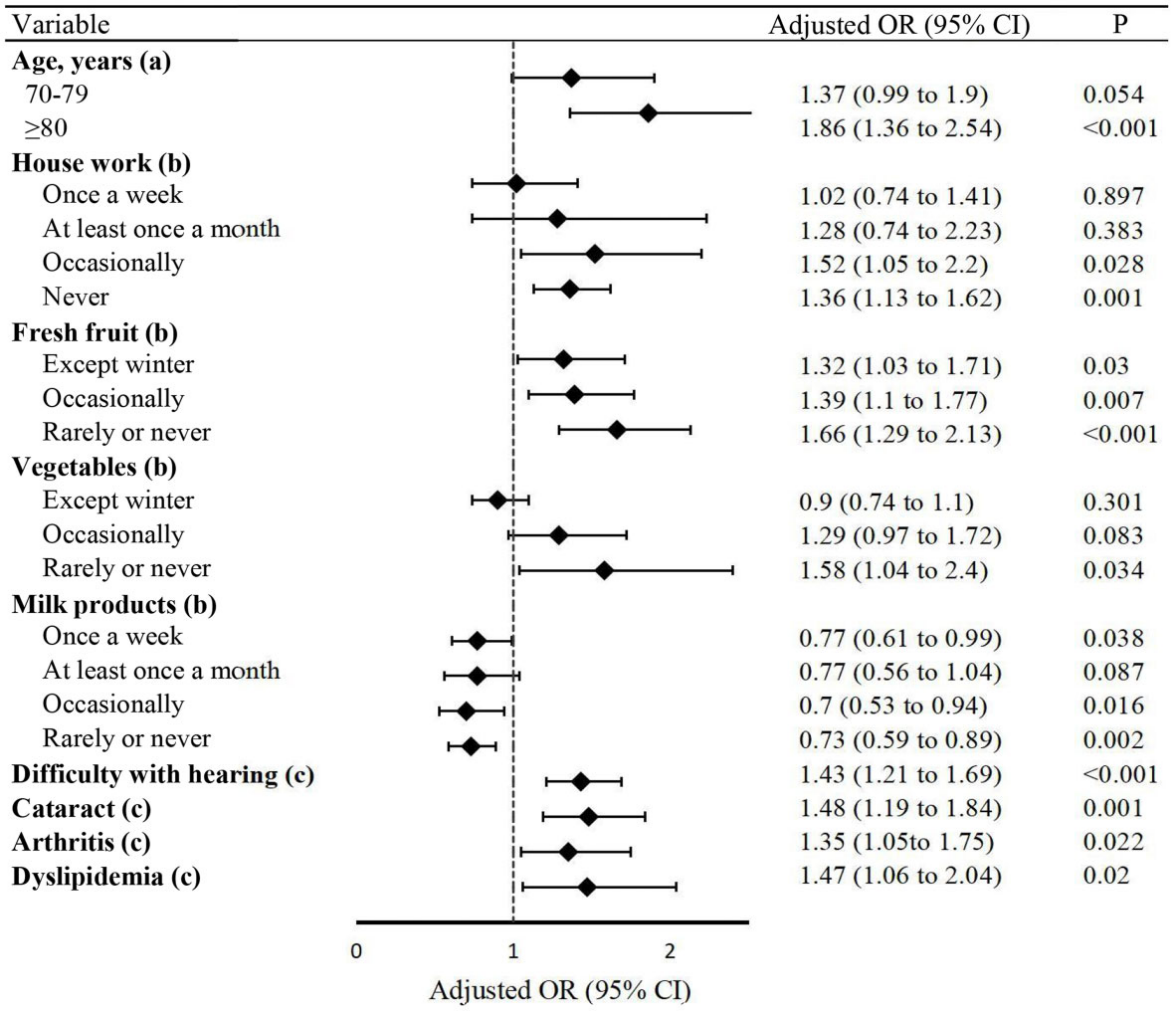
Forest plot showing the independent predictors of falls in older males (n=4555). OR: odds ratio; CI: confidence interval. (**a**) Age 65-69 years as a reference; (**b**) almost every day as a reference; (**c**) no event as a reference.

We conducted a logistic regression analysis of the potential factors that may increase the risk of falls in older men, and the results are shown in Supplementary Table S3 and Figure 2. Logistic regression revealed that the independent risk factors for falls in older men were age ≥80 years, occasionally or never doing housework, occasionally or never (or rarely) eating fresh fruits, never (or rarely) eating fresh vegetables, hearing difficulties, and having cataracts, arthritis, or dyslipidemia. Compared with the daily consumption of milk products, the consumption of milk products once a week or occasionally was a protective factor against falls in older men. Notably, never (or rarely) eating milk products was also a protective factor.

### Risk factors for falls in older women

**Figure 3:**
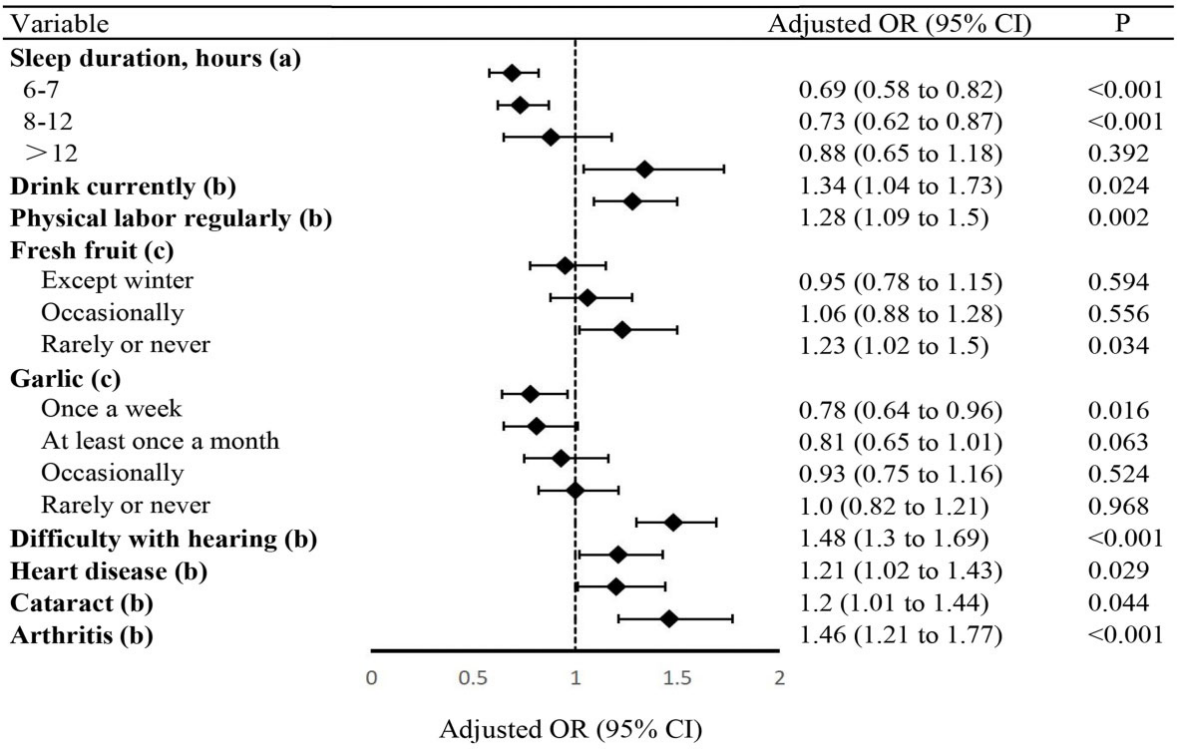
Forest plot showing the independent predictors of falls in older females (n=5182). OR: odds ratio; CI: confidence interval. (**a**) Less than 6 h of sleep duration as a reference; (**b**) no event as a reference; (**c**) almost every day as a reference.

The factors related to the risk of falls in older women suggested by the results of logistic regression are shown in Supplementary Table S4 and Figure 3. Our statistical results revealed that drinking alcohol, physical labor, never (or rarely), eating fresh fruit, hearing difficulties, heart disease, cataracts, and arthritis were associated with falls in older women. Sleeping 6-7 or 8-12 h a day and eating garlic once a week were protective factors.

## Discussion

This study investigated the prevalence of falls among older people in Chinese communities and the risk factors for falls among older men and women, including sociodemographic variables, living habits, eating habits, and health status factors. Our research showed that the prevalence of falls among older adults in China is high and that there are differences in independent risk factors between older men and women, which is conducive to accurate formulation of fall prevention strategies for communities or medical systems.

This study showed that the prevalence of falls among older adults in Chinese communities was 21.6%, among which the prevalence of falls in older women (24.6%) was significantly greater than that in men (18.1%). The epidemiological data published by Zhang et al. (9) shows that the prevalence of falls among urban older people in China is lower than that among rural people (15.0 *vs* 17.0%), while our data showed that the prevalence of falls among urban and rural older people was similar (21.8 *vs* 21.2%). Another study showed that in recent years, with the development of urbanization in China, the number of older people in urban areas has been continuously increasing (14), which may be the reason for the increase in falls among the older population compared to before (9). A survey showed that in 2018 the prevalence of falls among adults aged 65 years and older in the U.S. in the past year was 27.5% (15), which suggests that the prevalence of falls among older adults in China is lower than that in the U.S. but that the absolute number of falls among older adults in China is greater.

The risk of falls in older adults increases with age, which is also reflected in our research results. The data of this study showed that the prevalence of falls among older adults aged >80 years was 24.8%, which was much greater than that among older adults aged 65-69 years (14.2%). A previous study (16) reported that the prevalence of falls among older adults in rural areas (17%) is greater than that among older adults in urban areas (15%). However, the present study was based on the latest CLHLS data, and our research showed that the prevalence of falls among older adults in urban areas (22.6%) was greater than that in rural areas (21.2%). In addition, our study showed that the prevalence of falls among older adults living in institutions or in rehabilitation centers (26.2%) was greater than that among older adults living with family members (21.1%). Strengthening the attention and care given to older adults living in institutions may be the key to reducing the prevalence of falls among them.

Never (or rarely) eating fresh fruit, hearing difficulties, cataracts, and arthritis were common independent risk factors for falls among older adults in China, which was an important finding of this study. Sim et al. reported that there is no significant correlation between fruit intake and reduction in hospital fall rate (hazard ratio (HR)=1.03, 95%CI: 0.93 to 1.14) (17). Our research results showed that, compared with eating fresh fruit almost once a day, never (or rarely) eating fresh fruit can increase the risk of falls in older adults, which means that eating fresh fruit once a day can reduce the risk of falls. Research suggests that greater fruit intake is related to greater muscle strength and quality and physical function in older adults (17,18), which may explain the benefit of eating fresh fruits for fall prevention.

Hearing difficulties and cataracts are common conditions associated with older age, which means that older adults' perceptions of the external environment (vision and hearing) are weakened, thus increasing the risk of falls (19,20). The occurrence of arthritis, especially knee arthritis and hip arthritis, weakens older adults' ability to move and balance, which greatly exacerbates falls in older adults. The balance ability and activity level of patients with osteoarthritis or rheumatoid arthritis are significantly decreased (21-23), which directly increases the risk of falls in older adults.

With increasing age, the muscle strength and balance ability of older adults are significantly reduced, and these changes are accompanied by other chronic diseases (24-26), which directly or indirectly increase the risk of falls. At present, most epidemiological studies show that older adults are prone to falling when doing housework (27,28). However, this study showed that men who never performed housework were more likely to fall, which may be a sex-related difference in China. In China, women perform more housework than men do. We believe that because older Chinese men never perform housework, they will be unfamiliar with their living environment and will lack exercise, which may represent some of the reasons why they are prone to falling.

Dyslipidemia is closely related to hypertension, cognitive impairment, and cerebrovascular diseases (29,30), and these chronic diseases seriously affect the reaction ability and mobility of older adults. Eating milk products once a week can reduce the risk of falls in older men because regular consumption of milk products can increase the intake of calcium and protein (31). Interestingly, never eating milk products is also a protective factor, which warrants further exploration in future research.

Drinking alcohol, especially getting drunk, leads to significant limitations in behavior and judgment. Taylor et al. (32) conducted a meta-analysis on the relationship between alcohol consumption and accidental injuries (including falls) and found that the risk of accidents such as falls increased nonlinearly with increasing alcohol consumption. Physical labor may negatively affect the muscle strength and endurance of older women and further weaken their balance ability and mobility (33). Older people with heart disease are prone to a series of symptoms, such as dyspnea and chest pain, which may indirectly lead to falls (34). Aburub et al. (34) showed that older adults with cardiovascular diseases have a greater risk of falls. Many studies have confirmed that when older adults sleep less than 6 h, the occurrence and frequency of falls increase significantly (35,36). Studies suggest that sleeping less than 6 h can reduce the secretion of growth hormone (GH), insulin-like growth factor (IGF-1) and testosterone, which hinder muscle protein synthesis and increase the catabolism of skeletal muscle proteins (37,38). Our research showed that the best sleep duration for older adults was 6-12 h, which provides a basis for educating older adults on the best sleep duration. One study suggested that garlic can alleviate the progression of osteoarthritis and protect chondrocytes via the inhibition of the expression of matrix-degrading proteases in chondrocytes (39). Our research revealed that eating garlic once a week can reduce the risk of falls in older adults, but the underlying mechanism still needs to be further studied in multiple disciplines.

### Limitations

This study had several limitations. On the one hand, the survey data selected in this study are from a single cross-sectional study (2018 wave), and some variables that might influence the outcome were not measured, which makes it difficult to conduct a comprehensive discussion of more complex causal relationships. On the other hand, some of the variables we included were not precisely defined. For example, fresh fruit and vegetable consumption were not classified by specific type or intake. Solving these limitations in future research will help to further guide the accurate formulation of prevention strategies.

### Conclusion

The knowledge gained in this study regarding risk factors and protective factors can be used to inform community or medical system decisions when formulating reasonable recommendations for living habits, diet, and chronic disease control strategies to reduce the risk of falls in older adults.

## Supplementary Material

Click to view [pdf].
