# Supplementary material for: Prevalence and risk factors for falls among older Chinese adults in the community: findings from the CLHLS study
Source: Braz J Med Biol Res. 2024 May 17;57:e13469. doi: 10.1590/1414-431X2024e13469 (PMC11101163; doi:10.1590/1414-431X2024e13469)
Supplement: Supplementary file 1 [file 1414-431X-bjmbr-57-e13469-suppl.pdf]

Table S1. Univariate analysis of falls in older adults form the Chinese Longitudinal Healthy Longevity Survey (CLHLS, 2018 wave) (male and female).

Variables	Total (n=9737)	Non-Fallers (n=7636)	Fallers (n=2101)	t/χ2/Z	P
Age (years), mean (SD)	84.26 ± 11.4	83.57 ± 11.36	86.78 ± 11.2	–11.486	0.000
Age (n, %)				–9.981	0.000
<70	1091 (11.2)	936 (12.3)	155 (7.4)		
70–79	2590 (26.6)	2143 (28.1)	447 (21.3)		
≥80	6056 (62.2)	4557 (59.6)	1499 (71.3)		
Gender (n, %)				59.978	0.000
Male	4555 (46.8)	3729 (48.8)	826 (39.3)		
Female	5182 (53.2)	3907 (51.2)	1275 (60.7)		
BMI, mean (SD)	22.85 ± 8.96	22.9 ± 8.21	22.67 ± 11.26	1.069	0.285
BMI (n, %)				–4300	0.000
<18.5	1566 (16.1)	1165 (15.3)	401 (19.1)		
18.5–23.99	4998 (51.3)	3918 (51.3)	1080 (51.4)		
24–27.99	2347 (24.1)	1901 (24.9)	446 (21.2)		
≥28	826 (8.5)	652 (8.5)	174 (8.3)		
Residential area (n, %)				1.624	0.444
City	2167 (22.3)	1678 (22.0)	489 (23.3)		
Town	3263 (33.5)	2566 (33.6)	697 (33.2)		
Rural	4307 (44.2)	3392 (44.4)	915 (43.6)		
Co-residence (n, %)				7.219	0.027
With household member(s)	7884 (81.0)	6222 (81.5)	1662 (79.1)		
Alone	1586 (16.3)	1217 (15.9)	369 (17.6)		
In an institution	267 (2.7)	197 (2.6)	70 (3.3)		
Public old-age insurance (n, %)				5.141	0.023
No	6968 (71.6)	5506 (72.1)	1462 (69.6)		
Yes	2769 (28.4)	2130 (27.9)	639 (30.4)		
Waist circumference (cm), mean (SD)	84.69 ± 11.54	84.85 ± 11.48	84.08 ± 11.76	2.714	0.007
Hip circumference (cm), mean (SD)	91.82 ± 11.03	91.97 ± 11.04	91.27 ± 10.96	2.558	0.011
Sleep duration (hours, n, %)				–2.024	0.043
<6	1819 (18.7)	1333 (17.5)	486 (23.1)		
6–7	3337 (34.3)	2702 (35.4)	635 (30.2)		
8–12	4087 (42.0)	3238 (42.4)	849 (40.4)		
>12	494 (5.1)	363 (4.8)	131 (6.2)		
Smoke currently (n, %)				13.863	0.000
No	8158 (83.8)	6342 (83.1)	1816 (86.4)		
Yes	1579 (16.2)	1294 (16.9)	285 (13.6)		
Drink currently (n, %)				0.719	0.397
No	8224 (84.5)	6347 (84.3)	1718 (85.1)		
Yes	1513 (15.5)	1199 (15.7)	314 (14.9)		
Physical labor regularly (n, %)				3.379	0.066
No	2283 (23.4)	1822 (23.9)	461 (21.9)		
Yes	7454 (76.6)	5814 (76.1)	1640 (78.1)		
House work (n, %)				–7.101	0.000
Almost everyday	4420 (45.4)	3587 (47.0)	833 (39.6)		
Once a week	620 (6.4)	514 (6.7)	106 (5.0)		
At least once a month	158 (1.6)	126 (1.7)	32 (1.5)		
Occasionally	342 (3.5)	266 (3.5)	76 (3.6)		
Never	4197 (43.1)	3143 (41.2)	1054 (50.2)		
Tai chi chuan (n, %)				–2.634	0.008
Almost everyday	154 (1.6)	126 (1.7)	28 (1.3)		
Once a week	54 (0.6)	46 (0.6)	8 (0.4)		
At least once a month	24 (0.2)	23 (0.3)	1 (0.0)		
Occasionally	62 (0.6)	54 (0.7)	8 (0.4)		
Never	9443 (97.0)	7387 (96.7)	2056 (97.9)		
Square dance (n, %)				–0.909	0.364
Almost everyday	239 (2.5)	192 (2.5)	47 (2.2)		
Once a week	106 (1.1)	88 (1.2)	18 (0.9)		
At least once a month	63 (0.6)	49 (0.6)	14 (0.7)		
Occasionally	83 (0.9)	64 (0.8)	19 (0.9)		
Never	9246 (95.0)	7243 (94.9)	2003 (95.3)		
Garden work (n, %)				–0.834	0.404
Almost everyday	1225 (12.9)	982 (12.9)	273 (13.0)		
Once a week	248 (2.5)	204 (2.7)	44 (2.1)		
At least once a month	149 (1.5)	123 (1.6)	26 (1.2)		
Occasionally	167 (1.7)	133 (1.7)	34 (1.6)		
Never	7918 (81.3)	6194 (81.1)	1724 (82.1)		
Fresh fruit (n, %)				–5.649	0.000
Almost everyday	2157 (22.2)	1738 (22.8)	419 (19.9)		
Except winter	2397 (24.6)	1934 (25.3)	463 (22.0)		
Occasionally	2923 (30.0)	2288 (30.0)	635 (30.2)		
Rarely or never	2260 (23.2)	1676 (21.9)	584 (27.8)		
Vegetables (n, %)				–3.905	0.000
Almost everyday	6406 (65.8)	5082 (66.6)	1324 (63.0)		
Except winter	2378 (24.4)	1873 (24.5)	505 (24.0)		
Occasionally	675 (6.9)	496 (6.5)	179 (8.5)		
Rarely or never	278 (2.9)	185 (2.4)	93 (4.4)		
Sugar (n, %)				–2.479	0.013
Almost everyday	1158 (11.9)	868 (11.4)	290 (13.8)		
Once a week	1728 (17.7)	1339 (17.5)	389 (18.5)		

At least once a month	1194 (12.3)	947 (12.4)	247 (11.8)		
Occasionally	1747 (17.9)	1393 (18.2)	354 (16.8)		
Rarely or never	3910 (40.2)	3089 (40.5)	821 (39.1)		
Garlic (n, %)				–3.999	0.000
Almost everyday	1981 (20.3)	1560 (20.4)	421 (20.0)		
Once a week	2496 (25.6)	2017 (26.4)	479 (22.8)		
At least once a month	1565 (16.1)	1257 (16.5)	308 (14.7)		
Occasionally	1497 (15.4)	1160 (15.2)	337 (16.0)		
Rarely or never	2198 (22.6)	1642 (21.5)	556 (26.5)		
Milk products (n, %)				–2.696	0.007
Almost everyday	2356 (24.2)	1783 (23.3)	573 (27.3)		
Once a week	1575 (16.2)	1246 (16.3)	329 (15.7)		
At least once a month	883 (9.1)	693 (9.1)	190 (9.0)		
Occasionally	1182 (12.1)	961 (12.6)	221 (10.5)		
Rarely or never	3741 (38.4)	2953 (38.7)	788 (37.5)		
Nut products (n, %)				–4.699	0.000
Almost everyday	608 (6.2)	484 (6.3)	124 (5.9)		
Once a week	1203 (12.4)	985 (12.9)	218 (10.4)		
At least once a month	1062 (10.9)	851 (11.1)	211 (10.0)		
Occasionally	1653 (17.0)	1328 (17.4)	325 (15.5)		
Rarely or never	5211 (53.5)	3988 (52.2)	1223 (58.2)		
Water (n, %)				1.800	0.180
Boiled	9545 (98.0)	7493 (98.1)	2052 (97.7)		
Un-boiled	192 (2.0)	143 (1.9)	49 (2.3)		
Difficulty with hearing (n, %)				115.767	0.000
No	6087 (62.5)	4985 (65.3)	1102 (52.5)		
Yes	3650 (37.5)	2651 (34.7)	999 (47.5)		
Hypertension (n, %)				2.943	0.086
No	5711 (58.7)	4513 (59.1)	1198 (57.0)		
Yes	4026 (41.3)	3123 (40.9)	903 (43.0)		
Diabetes (n, %)				1.444	0.229
No	8825 (90.6)	6935 (90.8)	1890 (90.0)		
Yes	912 (9.4)	701 (9.2)	211 (10.0)		
Heart disease (n, %)				9.128	0.003
No	8118 (83.4)	6412 (84.0)	1706 (81.2)		
Yes	1619 (16.6)	1224 (16.0)	395 (18.8)		
Tuberculosis (n, %)				3.741	0.053
No	9666 (99.3)	7587 (99.4)	2079 (99.0)		
Yes	71 (0.7)	49 (0.6)	22 (1.0)		
Cataract (n, %)				39.980	0.000
No	8428 (86.6)	6697 (87.7)	1731 (82.4)		
Yes	1309 (13.4)	939 (12.3)	370 (17.6)		
Glaucoma (n, %)				2.876	0.09
No	9545 (98.0)	7495 (98.2)	2050 (97.6)		
Yes	192 (2.0)	141 (1.8)	51 (2.4)		
Arthritis (n, %)				36.563	0.000
No	8714 (89.5)	6909 (90.5)	1805 (85.9)		
Yes	1023 (10.5)	727 (9.5)	296 (14.1)		
Dementia (n, %)				17.806	0.000
No	9606 (98.7)	7553 (98.9)	2053 (97.7)		
Yes	131 (1.3)	83 (1.1)	48 (2.3)		
Dyslipidemia (n, %)				10.024	0.002
No	9205 (94.5)	7248 (94.9)	1957 (93.1)		
Yes	532 (5.5)	388 (5.1)	144 (6.9)		
Chronic nephritis (n, %)				5.725	0.017
No	9627 (89.9)	7560 (99.0)	2067 (98.4)		
Yes	110 (1.1)	76 (1.0)	34 (1.6)		
Hepatitis (n, %)				6.597	0.01
No	9698 (99.6)	7612 (99.7)	2086 (99.3)		
Yes	39 (0.4)	24 (0.3)	15 (0.7)		

Data are reported as mean and SD or number and percentage.

Table S2. Multivariate logistic regression analysis in older adults form the Chinese Longitudinal Healthy Longevity Survey (CLHLS) (2018 wave).

Factors	β	Standard Error	Wald	OR (95%CI)	P
Age (years)	–	–	20.276	–	0.000
<70	–	–	–	1.00	–
70–79	0.177	0.103	2.977	1.194 (0.976 to 1.459)	0.084
≥80	0.392	0.099	15.639	1.481 (1.219 to 1.798)	0.000
Gender (male *vs* female)	0.33	0.052	39.998	1.391 (1.255 to 1.540)	0.000
Public old-age insurance	0.112	0.055	4.103	1.119 (1.004 to 1.247)	0.043
Sleep duration (hours)	–	–	21.410	–	0.000
<6	–	–	–	1.00	–
6–7	–0.306	0.071	18.657	0.736 (0.641 to 0.846)	0.000
8–12	–0.257	0.068	14.544	0.773 (0.677 to 0.882)	0.000
>12	–0.122	0.118	1.064	0.885 (0.702 to 1.160)	0.302
House work	–	–	10.817	–	0.029
Almost everyday	–	–	–	1.00	–
Once a week	–0.098	0.116	0.717	0.906 (0.722 to 1.138)	0.397
At least once a month	0.090	0.206	0.192	1.095 (0.731 to 1.639)	0.661
Occasionally	0.157	0.139	1.271	1.170 (0.890 to 1.538)	0.260
Never	0.168	0.06	7.838	1.182 (1.052 to 1.330)	0.005
Fresh fruit	–	–	25.773	–	0.000
Almost everyday	–	–	–	1.00	–
Except winter	0.071	0.078	0.839	1.074 (0.922 to 1.251)	0.360
Occasionally	0.196	0.075	6.851	1.216 (1.050 to 1.408)	0.009
Rarely or never	0.363	0.078	21.723	1.437 (1.234 to 1.671)	0.000
Garlic	–	–	12.315	–	0.015
Almost everyday	–	–	–	1.00	–
Once a week	–0.166	0.077	4.672	0.847 (0.729 to 0.985)	0.031
At least once a month	–0.145	0.086	2.817	0.865 (0.730 to 1.025)	0.093
Occasionally	–0.031	0.085	0.135	1.032 (0.873 to 1.219)	0.714
Rarely or never	0.038	0.077	0.239	1.038 (0.893 to 1.208)	0.625
Milk products	–	–	15.652	–	0.004
Almost everyday	–	–	–	1.00	–
Once a week	–0.171	0.081	4.434	0.843 (0.719 to 0.988)	0.035
At least once a month	–0.093	0.099	0.893	0.911 (0.750 to 1.106)	0.345
Occasionally	–0.306	0.093	10.847	0.736 (0.614 to 0.883)	0.001
Rarely or never	–0.222	0.068	10.788	0.801 (0.701 to 0.914)	0.001
Difficulty with hearing	0.338	0.055	37.440	1.403 (1.259 to 1.563)	0.000
Cataract	0.232	0.071	10.640	1.261 (1.097 to 1.449)	0.001
Arthritis	0.347	0.078	19.602	1.415 (1.214 to 1.650)	0.000
Dyslipidemia	0.265	0.108	6.028	1.304 (1.055 to 1.611)	0.014
Hepatitis	0.857	0.345	6.174	2.355 (1.198 to 4.629)	0.013

Table S3. Multivariate logistic regression analysis of falls in male older adults form the Chinese Longitudinal Healthy Longevity Survey (CLHLS) (2018 wave).

Factors	β	Standard Error	Wald	Adjusted OR (95%CI)	P
Age (years)	–	–	19.660	–	0.000
<70	–	–	–	1.00	–
70–79	0.317	0.165	3.705	1.373 (0.994 to 1.897)	0.054
≥80	0.621	0.160	15.104	1.860 (1.360 to 2.543)	0.000
House work	–	–	13.641	–	0.009
Almost everyday	–	–	–	1.00	–
Once a week	0.021	0.164	0.017	1.021 (0.741 to 1.408)	0.897
At least once a month	0.247	0.283	0.762	1.280 (0.735 to 2.229)	0.383
Occasionally	0.417	0.190	4.831	1.517 (1.046 to 2.201)	0.028
Never	0.304	0.093	10.739	1.355 (1.130 to 1.624)	0.001
Fresh fruit	–	–	15.922	–	0.001
Almost everyday	–	–	–	1.00	–
Except winter	0.281	0.129	4.730	1.324 (1.028 to 1.705)	0.030
Occasionally	0.332	0.122	7.345	1.393 (1.096 to 1.771)	0.007
Rarely or never	0.507	0.128	15.816	1.661 (1.293 to 2.132)	0.000
Vegetables	–	–	9.350	–	0.025
Almost everyday	–	–	–	1.00	–
Except winter	–0.104	0.101	1.071	0.901 (0.740 to 1.098)	0.301
Occasionally	0.253	0.146	2.999	1.288 (0.967 to 1.716)	0.083
Rarely or never	0.454	0.214	4.490	1.575 (1.035 to 2.398)	0.034
Milk products	–	–	11.312	–	0.023
Almost everyday	–	–	–	1.00	–
Once a week	–0.258	0.125	4.286	0.773 (0.605 to 0.986)	0.038
At least once a month	–0.268	0.157	2.921	0.765 (0.562 to 1.040)	0.087
Occasionally	–0.350	0.145	5.854	0.704 (0.530 to 0.936)	0.016
Rarely or never	–0.322	0.105	9.404	0.725 (0.590 to 0.890)	0.002
Difficulty with hearing	0.358	0.085	17.748	1.431 (1.211 to 1.691)	0.000
Cataract	0.389	0.112	12.068	1.475 (1.185 to 1.838)	0.001
Arthritis	0.303	0.132	5.250	1.353 (1.045 to 1.753)	0.022
Dyslipidemia	0.387	0.167	5.401	1.473 (1.062 to 2.041)	0.020

Table S4. Multivariate logistic regression analysis of falls in female older adults form the Chinese Longitudinal Healthy Longevity Survey (CLHLS) (2018 wave).

Factors	β	Standard Error	Wald	OR (95%CI)	P
Sleep duration (hours)	–	–	20.463	–	0.000
<6	–	–	–	1.00	-
6–7	–0.375	0.089	17.720	0.687 (0.577 to 0.818)	0.000
8–12	–0.311	0.086	13.155	0.733 (0.619 to 0.867)	0.000
>12	–0.131	0.153	0.732	0.878 (0.651 to 1.184)	0.392
Drink currently	0.292	0.129	5.126	1.339 (1.040 to 1.725)	0.024
Physical labor regularly	0.248	0.081	9.362	1.281 (1.093 to 1.502)	0.002
Fresh fruit	–	–	8.566	–	0.036
Almost everyday	–	–	–	1.00	–
Except winter	–0.054	0.100	0.284	0.948 (0.778 to 1.154)	0.594
Occasionally	0.056	0.095	0.347	1.058 (0.878 to 1.275)	0.556
Rarely or never	0.210	0.099	4.493	1.233 (1.016 to 1.497)	0.034
Garlic	–	–	10.290	–	0.036
Almost everyday	–	–	–	1.00	–
Once a week	–0.245	0.102	5.774	0.782 (0.641 to 0.956)	0.016
At least once a month	–0.211	0.113	3.446	0.810 (0.649 to 1.012)	0.063
Occasionally	–0.071	0.111	0.405	0.932 (0.749 to 1.159)	0.524
Rarely or never	–0.004	0.098	0.002	0.996 (0.821 to 1.208)	0.968
Difficulty with hearing	0.393	0.068	33.184	1.481 (1.296 to 1.693)	0.000
Heart disease	0.189	0.087	4.753	1.209 (1.019 to 1.433)	0.029
Cataract	0.183	0.091	4.039	1.201 (1.005 to 1.437)	0.044
Arthritis	0.379	0.097	15.311	1.462 (1.209 to 1.768)	0.000
